# Contribution of Population Pharmacokinetics of Glycopeptides and Antifungals to Dosage Adaptation in Paediatric Onco-hematological Malignancies: A Review

**DOI:** 10.3389/fphar.2021.635345

**Published:** 2021-04-01

**Authors:** Stéphanie Leroux, Françoise Mechinaud-Heloury, Evelyne Jacqz-Aigrain

**Affiliations:** ^1^ Department of Paediatrics, CHU Rennes, University of Rennes 1, Rennes, France; ^2^ Department of Paediatric Pharmacology and Pharmacogenetics, University Hospital Robert Debré (APHP), Rennes, France; ^3^ Department of Paediatric Hematology-Immunology, University Hospital Robert Debré (APHP), Paris, France; ^4^ Paris University, Paris, France

**Keywords:** paediatrics, malignancy, onco-hematology, glycopeptides, antifungals, population pharmacokinetics, drug dosage

## Abstract

The response to medications in children differs not only in comparison to adults but also between children of the different age groups and according to the disease. This is true for anti-infectives that are widely prescribed in children with malignancy. In the absence of pharmacokinetic/pharmacodynamic paediatric studies, dosage is frequently based on protocols adapted to adults. After a short presentation of the drugs, we reviewed the population pharmacokinetic studies available for glycopeptides (vancomycin and teicoplanin, *n* = 5) and antifungals (voriconazole, posaconazole, and amphotericin B, *n* = 9) currently administered in children with onco-hematological malignancies. For each of them, we reported the main study characteristics including identified covariates affecting pharmacokinetics and proposed paediatric dosage recommendations. This review highlighted the very limited amount of data available, the lack of consensus regarding PK/PD targets used for dosing optimization and regarding dosage recommendations when available. Additional PK studies are urgently needed in this specific patient population. In addition to pharmacokinetics, efficacy may be altered in immunocompromised patients and prospective clinical evaluation of new dosage regimen should be provided as they are missing in most cases.

## Introduction

Child specific challenges of treatment include 1) frequent off-label/unlicensed use (which increases the risk of adverse drug reactions and lack of efficacy), 2) limited pharmacokinetic (PK) and/or pharmacodynamic (PD) age group and disease specific data, 3) considerable variation in drug dosages, and 4) an increased risk of medication errors (Gore et al., 2017; Jong, et al., 2001).

According to pharmacoepidemiologic studies, anti-infective drugs are widely prescribed in children (de Bie et al., 2016) but paediatric pharmacokinetic/pharmacodynamic (PK/PD) studies are limited and dosage frequently based on protocols adapted to adults. However, the response of children to medication differs in comparison to adults and also between different paediatric age groups and between diseases. Data are also missing in many paediatric subpopulations presenting with frequent specific diseases.

In the past 20 years, several initiatives were undertaken in the United States and in Europe to encourage paediatric research and drug development (Choonara, 2007). Although the number of paediatric investigation plans and marketing authorisations increased, studies evaluating dosage regimen and therapeutic strategies in children presenting with frequent specific conditions are sparse and require public-private fundings (Turner et al., 2014; Ruggieri et al., 2015). This is particularly true in paediatric malignancies, representing specific diseases different from adults.

We selected paediatric onco-hematologic malignancies as a key area where anti-infectives are used regularly to manage infections complicating chemotherapy. However, dosage recommandations based on PK/PD targets originate frequently for adult studies. In this context, we reviewed the population PK (PopPK) studies available for currently prescribed glycopeptides (vancomycin and teicoplanin) and antifungals (posaconazole, voriconazole, and amphotericin B) to report on the key variables impacting PK parameters in children and analyze available dosage recommendations.

## Pharmacokinetic/Pharmacodynamic Biomarkers of Anti-Infectives

PK-PD indices represent the quantitative relationship between pharmacokinetic measures the test drug (such as area under the curve AUC) and a microbiologic measure of susceptibility (minimum inhibitory concentration - MIC). The microbiological data from animal or *in vitro* experiments provide initial insight into PK-PD most likely to be associated with efficacy.

A concentration-dependent pattern of activity may be observed and AUC_0-24_/MIC ratio and/or the Cmax/MIC ratio are the PK/PD indices that usually predict efficacy in PK-PD models.

A time-dependent pattern of bactericidal activity may be observed and time > MIC and/or AUC_0–24_/MIC ratio are the indices that usually predict efficacy in PK-PD models.

Glycopeptides are time-dependent/concentration-independent antibiotics with moderate persistent killing, active against susceptible strains of methicillin-resistant (beta-lactam resistant) staphylococci. For azole antifungals, the PK/PD index that best relates to the outcome is the AUC_0–24_/MIC (European Medicines Agency, 2015; Gómez-López, 2020).

## Presentation of Pharmacological Properties and Available PK Data of Glycopeptides and Antifungals

### Glycopeptides: Vancomycin and Teicoplanin

The first line treatment for invasive methicillin-resistant *Staphylococcus aureus* (MRSA) infections is a glycopeptide antibiotic, either vancomycin (a glycopeptide) or teicoplanin (a lipoglycopeptide). Both are often prescribed to broaden initial empirical antibiotic in case of persistent fever in paediatric and adult patients with HM (Libuit et al., 2014; Lehrnbecher et al., 2017). Teicoplanin is not inferior to vancomycin with regard to efficacy and is associated with a lower adverse event rate than vancomycin (Menichetti et al., 1994; Finch and Eliopoulos, 2005).

#### Vancomycin

Vancomycin is a large, hydrophilic molecule with poor oral absorption. Hence it is given intravenously to treat systemic infections. Vancomycin is 25–50% protein-bound, mainly to albumin and immunoglobulins, and protein binding changes non-linearly with vancomycin concentrations. It is almost exclusively eliminated by the renal route via glomerular filtration and to some extent via active tubular secretion. Elimination half-life is 6–12 h. Factors that affect its clinical activity, include variable tissue distribution, inoculum size, and emerging resistance.

The AUC_0–24_/MIC ratio is the best predictor of vancomycin efficacy in adults. Various studies have shown that a vancomycin AUC_0–24_/MIC ratio >400 best predicts treatment outcomes for invasive MRSA infection in adults.

Many vancomycin PK studies and reviews are available in adults, including PopPK studies and report that both vancomycin clearance (CL) and volume of distribution were higher in cancer than non-cancer patients (Yasuhara et al., 1998; Buelga et al., 2005; Jarkowski et al., 2012). PK studies also reported large variability in vancomycin disposition in children (neonates excluded) (Chang, 1995; Krivoy et al., 1998; Wrishko et al., 2000; Marsot et al., 2012; Hadi et al., 2016; Tkachuk et al., 2018).

#### Teicoplanin

Similarly to vancomycin, teicoplanin needs to be administered intravenously as bioavailability is extremely low, it is 25–50% protein bound and also exclusively eliminated by the renal route (Marsot et al., 2012).

PopPK studies available in adults receiving the drug for various indications, showed high variability in drug disposition (Yu et al., 1995; Lortholary et al., 1996; Soy et al., 2006). In children, different classical PK studies but with a relatively low number of patients (12,13, and 6, respectively, in the three studies) provided conflicting conclusions on the impact of age on teicoplanin PK parameters (Tarral et al., 1988; Terragna et al., 1988; Reed et al., 1997).

### Antifungals: Voriconazole, Posaconazole, and Amphotericin B

Invasive fungal disease (IFD) is an important cause of morbidity and mortality in immunocompromised children receiving chemotherapy for cancer and those undergoing hematopoietic stem cell transplant (HSCT). Its incidence varies according to chemotherapy regimen and supportive care practices (Lehrnbecher et al., 2009; Groll et al., 2014).

Antifungal chemoprophylaxis is therefore recommended for high risk patients with prolonged neutropenia, prolonged use of steroids or in different subgroups of leukemia, taking into account the local epidemiology, patient comorbidity or specific treatment modalities (Fisher et al., 2018).

There are no major differences between children and adults in the choice of treatment of established infections and triazole antifungal agents are potential choices both for prophylaxis and treatment of probable and proven IFDs.

#### Voriconazole

Voriconazole is a recent triazole antifungal agent with potent activity against a wide range of clinically significant pathogens, including Aspergillus and Candida. Voriconazole may be administered orally and intravenously. Bioavailability has been estimated to be >90% in healthy volunteers, substantially lower in adults and even more in children with malignancies (Karlsson et al., 2009). A potential mechanistic explanation could be that paediatric patients exhibit greater systemic metabolism but also greater first-pass metabolism than that of adults.

Voriconazole is extensively metabolized by polymorphic cytochrome P450 (CYP) isoenzymes CYP2C9, CYP2C19 and CYP3A4. The prevalence of CYP2C19 poor metabolizers is 3–5% among Caucasians and black Africans, 15–20% among Asian populations. Only <2% of the dose is excreted unchanged in the urine. AUC/MIC ratio >20–25 was previously proposed as the PK/PD target to optimize voriconazole dosing (Andes, 2003). Previously published reports in adult patients suggested aiming at a plasma trough drug concentration between 1 and 5.5 mg/L for efficacy and limiting toxicity (Pascual et al., 2008). PopPK studies on voriconazole have been conducted in adults, either healthy volunteers or sick patients (Vehreschild et al., 2012; van Iersel et al., 2018; Liu et al., 2019; Shi et al., 2019) and available studies were recently reviewed (Shi et al., 2019), reporting marked IIV in PK parameters and only limited intraindividual variation.

#### Posaconazole

Posaconazole is licensed for prophylaxis of IFD in 1) patients with prolonged neutropenia and who are at high risk of developing IFD complicating HM, 2) patients at high risk of developing IFD following HSCT and under immunosuppressive therapy for graft-versus-host disease.

Posaconazole showed potent dose-dependent *in vivo* antifungal activity on prophylaxis and treatment against most fungal infections. PK studies were predominantly performed in healthy volunteers and hematological adult patients and were recently reviewed (Chen et al., 2019). Bioavailability was reported to be around 50% in healthy volunteers with the suspension and delayed-release tablet (Chen et al., 2019), but lower in patients receiving the posaconazole suspension (Dolton et al., 2014). It is bound to plasma proteins for more than 98%. It is predominantly eliminated unchanged in feces or in urine. Elimination by glucuronidation (UGT1A4) is only limited (less than 20%).

Different biomarkers of efficacy are reported in adults, 1) the AUC/MIC showed the strongest correlation with therapeutic success, 2) the posaconazole average plasma concentration (Cavg) ≥ 1.25mg/L at steady-state was fixed as a valid cut-off value for IFD treatment as it was associated with 75% successful response rates in patients with invasive aspergillosis (Chen et al., 2020), while Cavg of 0.7 mg/L is accepted as a target for prophylaxis. Trough concentrations (Cmin) proved to be well correlated with Cavg or AUC 0–24 are biomarkers easier to use for monitoring.

According to the European marketing authorization, safety and efficacy are not established in children aged below 18 years (Table 1). Posaconazole can be administered as an oral suspension (40 mg/ml), a delayed-release tablet (100 mg), and more recently as intravenous formulation (18 mg/ml): only the oral formulations are licensed for paediatric patients. Prophylactic posaconazole was shown superior to fluconazole or itraconazole in reducing IFD and fungal related mortality in patients with graft vs. host disease (Ullmann et al., 2007). In addition, acceptability is high. Dosing information depends on the formulation, patient’s age and indication (prophylaxis or treatment). No PK/PD data exist on tablets and intravenous formulation for all paediatric age groups.

**TABLE 1:** Indications and European marketing authorization status of glycopeptides and antifungals in the different paediatric age groups.

Drug	Indications	Marketing authorization in Europe
Glycopeptides		
Vancomycin	Serious infections due to Gram-positive bacteria such as methicillin-resistant staphylococcus aureus (MRSA), resistant to other antibiotics	All patients; dosage based on age and weight
Teicoplanin	Serious infections due to Gram-positive bacteria	All patients; dosage based on age and weight
**Antifungals**		
Voriconazole	Treatment of	Adults and children aged 2 years and above
	1) invasive aspergillosis	
	2) candidemia in non-neutropenic patients	
	3) fluconazole-resistant serious invasive Candida infections (including C. krusei)	
	4) serious fungal infections caused by Scedosporium spp. and Fusarium spp.	
	Prophylaxis of invasive fungal infections in high risk allogeneic hematopoietic stem cell transplant recipients	
Posaconazole	Treatment of	Safety and efficacy not established in children aged below 18 years
	1) invasive aspergillosis in patients with disease that is refractory or intolerant to amphotericin B or itraconazole	
	2) oropharyngeal candidiasis: as first-line therapy in patients who have severe disease or are immunocompromised	
	Prophylaxis of invasive fungal infections	
	1) in patients receiving chemotherapy for acute myelogenous leukemia or myelodysplastic syndromes at risk of prolonged neutropenia and invasive fungal infections	
	2) in hematopoietic stem cell transplant recipients under high-dose immunosuppressive therapy for graft-versus-host disease, at high risk of invasive fungal infections	
Amphotericin B–lipid formulation	1) Systemic mycotic infections due to susceptible organisms	Patients who are one month to 18 years old; dosage based on weight
	2) Fever of unknown origin in neutropenic patients	

#### Amphotericin B

Amphotericin B is a highly lipophilic drug administered intravenously as it is poorly absorbed orally. Amphotericin B-deoxycholate (D-AmB), a mixed micellar dispersion with deoxycholate, has been the cornerstone for the management of life-threatening fungal infections. This formulation has suboptimal clinical success and frequent nephron-toxic effects at usual recommended doses. It was replaced in the 1990 by the lipid-based formulations (L-AmB) encapsulating amphotericin B into liposomes or binding the drug to lipids (Walsh et al., 1999; Chen et al., 2020). L-AmB is less toxic and has limited infusion-associated reactions, increased therapeutic index and can be administered at higher dosages (Janoff, 1990; Moreau et al., 1992). AmB exhibits concentration-dependent killing of fungal organisms, with a long post-antifungal effect (Andes et al., 2003).

Standard PK studies, comparing the pharmacokinetics and tolerability of amphotericin B administered in a conventional 5% dextrose (glucose) (5% D) solution and in a 20% fat emulsion formulation (Intralipid; 20% IL) were initially conducted in adults. Differences in PK profile between the two formulations resulted in higher distribution volume, decreased Cmax and AUC, and increased CL with the 20% IL form (Ayestarán et al., 1996). A possible reason for the PK differences between the two methods of administration is the larger particle size of AmB in lipid emulsion.

The first PK studies in neutropenic adults showed dose-related, non-linear, saturation-like PK. The Cmax/MIC ratio of 2 may provide sufficient antifungal efficacy. The first population PK in adults (75 patients received 0.5–8.0 mg/kg of body weight of amphotericin B colloidal dispersion for 28 days) showed that plasma CL and volume of distribution increased with escalating doses, but without net change in renal function (Amantea et al., 1995).

## Review of the Population Pharmacokinetic Studies of Glycopeptides and Antifungals in Paediatric Onco-hematological Malignancies

### Methods

We search for PopPK studies of anti-infectives (glycopeptides and antifungals) in onco-hematological malignancies (leukemia or lymphoma or multiple myeloma or malignant disease) affecting paediatric patients (neonates excluded), published up to August 31, 2020, using Pubmed to identify the PopPK studies of glycopeptides/vancomycin/teicoplanin, antifungals/voriconazole/posaconazole/amphotericinB, in children/paediatric patients with hemato-oncology malignancy/acute leukemia/lymphoma. We selected the studies on one of our five anti-infectives of interest and selected additional articles by reviewing the bibliography of the selected publications. Tables were built to summarize the PopPK studies, presenting the study drug, patients’ characteristics (number, age, and weight), underlying disease and indication of treatment, number of samples, software, covariates analyzed, and retained in the PK model, PK/PD target used for simulations and dosage recommendations.

### Results

#### Studies Selection

A total of 19 paediatric PopPK studies were identified and 14 included in this review: five for glycopeptides (vancomycin *n* = 3 and teicoplanin *n* = 2) and 9 for antifungals (voriconazole *n* = 4, posaconazole *n* = 1, amphotericin B *n* = 4) administered in children with malignancy.

#### Population Pharmacokinetics of Glycopeptides in Paediatric Onco-hematological Malignancies

The PopPK studies of glycopeptides in paediatric HM are presented in Table 2.

**TABLE 2:** Population pharmacokinetic studies of glycopeptides in paediatric onco-hematological malignancies.

	Vancomycin		Teicoplanin	
Author	Zhao 2014 Zhao et al. (2014)	Guilhaumou 2016 and Marsot 2018 Marsot et al. (2018), Guilhaumou et al. (2016)	Ramos 2014 Ramos-Martín et al. (2014)	Zhao 2015 Zhao et al. (2015)
Study location	France	France	United Kingdom	France
Underlying disease (number of patients)	HM including	Malignant diseases including	Predominantly malignant diseases (not detailed)	HM including
	- ALL n=64	- HM n=32		- ALL n=65
	- AML N=48	- SM n=30		- AML n=27
Indication	Suspected or proven infection	Suspected infection (Febrile neutropenia)	At the discretion of the treating physician	Suspected infection
Number of patients	70	121	39	85
Age (years) mean ± SD	6.8 ± 4.8	HM: 9.1 ± 5.7	4 ± 4.3	8.4 ± 4.6
		SM: 7.1 ± 5.4	
Weight (kg) *mean ± SD*	25.7 ± 15.5	HM: 31.6 ± 18.6	17.3 ± 13.3	32.3 ± 17.8
		SM: 25.0 ± 16.4	
Intravenous drug dose	40–60 mg/kg/24 h in four divided doses (over 1 h)	10–15 mg/kg LD (over 1 h) followed by MD 30–40 mg/kg/24 h continuous infusion	10 mg/kg BID for 3 LD followed by MD 10 mg/kg/24 h (“current dosage”)	10 mg/kg BID for 3 LD (over 3–5 min) followed by MD 10 mg/kg/24 h
PK sampling design	TDM sampling	TDM sampling	Specific PK study sampling	TDM and opportunistic sampling
Number of samples	98	301	298	143
Software	NONMEM	NONMEM	Pmetrics	NONMEM
Number of compartment(s)	1	1	2	2
Significant covariate on CL	WT (Alloestcoef function, MEDcentred), CrCL	WT (Allofixcoef function, 70 kg centred)	WT (linear function, noncentred)	WT (Allofixcoef function, MEDcentred), CrCL
		Type of disease (HM or SM)	
		Cyclosporin coadministration in case of HM	
Significant covariate on V	WT (Alloestcoef function, MEDcentred)	None	None	WT (Allofixcoef function, MEDcentred)
Covariates tested without significant effect on PK parameters	Age, serum creatinine, type of disease (leukaemia or lymphoma), and bone marrow transplantation	Age, gender, serum creatinine, and comedications (acyclovir, aminoglycoside, foscavir, and liposomal amphotericin b)	Height, age, serum creatinine, and comedications	Age, serum creatinine, and type of disease (leukaemia or lymphoma)
CL estimates	Typical value: 4.3 L/h for a patient weighing 20 kgMean (range) individual values: 0.22 (0.04–0.73) L/h/kg	Typical value: 4.7 L/h standardised to a 70 kg individual with HM and without cylcosporin coadministration Mean individual value: 0.084 L/h/kg	Median individual value: 0.019 L/h/kg	Median individual values (L/h/kg): Infants: 0.028, Children: 0.019 Adolescents: 0.015
Validation	Internal and External (20 children, 25 samples)	Internal and External (77 children, 289 samples)	Internal	Internal and External (15 children, 15 samples)
PK/PD target used for dosing optimization (simulations)	1) AUC_0-24_/MIC ≥400 h	SS concentrations of 20–25 mg/L	C_min_ > 10 mg/L	1) AUC_0-24_: 750 mg/h/l
	2) C_min_ of 10–20 mg/L at SS			2) C_min_ of 10–30 mg/L at SS
Dosage recommendation based on results of modelling and simulation	Patient tailored dose based on WT and CrCL	Chart based on WT and coadministration of cyclosporine (administration via continuous infusion after a LD of 15 mg/kg)	“Current dosage” based on WT is adequate but TDM is highly recommended	Patient tailored dose based on WT and CrCL

HM, hematological malignancy; ALL, acute lymphoblastic leukaemia; AML, acute myeloid leukaemia; SM, solid malignancy; LD, loading dose; MD, maintenance dose; PK, pharmacokinetic; TDM, therapeutic drug monitoring; CL, clearance; V, volume of distribution; WT, weight; Allofixcoef function, weight included as an allometric power function using fixed coefficients of 0.75 for CL and one for V; Alloestcoef function, weight included as an allometric power function using estimated coefficients for CL and V; MEDcentred, centred on the median weight of the population; 70kgcentred, normalized according to data for a 70kg individual; CrCL, creatinine clearance; Internal validation, diagnostic plots +/− visual predictive checks +/− bootstrap +/− NPDE +/− weighted-mean error and bias-adjusted weighted-mean-squared error; PD, pharmacodynamic; AUC area under the concentration-time curve; MIC, minimum inhibitory concentration; C_min_, trough concentration; SS, steady-state.

During our review process, two vancomycin PopPK studies in sick children were excluded as the underlying disease and indications of treatment were missing, or patients with various diseases were included (Lamarre et al., 2000; Hahn et al., 2015). We identified and analyzed three vancomycin PopPK studies (Zhao et al., 2014; Marsot et al., 2018; Guilhaumou et al., 2016) including two PopPK studies in paediatric HM and 1 being an external validation (Guilhaumou et al., 2016) of one of them (Marsot et al., 2018). Data are presented in Table 2. The number of patients included were 70 (Zhao et al., 2014) and 121 (Marsot et al., 2018), with a wide range of both ages and weights. Concentrations were obtained during therapeutic drug monitoring (TDM). Among all covariates tested, weight (with fixed or estimated allometric coefficients) was always significant, creatinine CL was significant only in the model by Zhao (Zhao et al., 2014), type of disease and coadministration of cyclosporin were significant only in the model by Guilhaumou (Guilhaumou et al., 2016). The primary PK/PD target used for simulations was AUC/MIC ≥400 h in one case and the steady-state concentration of 20–25 mg/L in the other one. After accounting for significant covariates, the mean value of the interindividual variability (IIV) in vancomycin CL was 34.8 and 31.1%, respectively, in the two studies. Both studies showed that higher doses are required in cancer paediatric patients. Dosage adaptation might use either a patient tailored dose or a proposed chart, taking into account the identified variables. In both cases, drug monitoring is still recommended.

We identified and analyzed two teicoplanin PopPK studies in children with cancer (Ramos-Martín et al., 2014; Zhao et al., 2015). Two additional studies population PK studies are available but in children without malignancy treated in intensive care or with renal dysfunction (Lukas et al., 2004; Gao et al., 2020). Results of the analyzed PopPK studies showed that weight and renal function (quantified by creatinine CL) are covariates explaining part of the observed variability. PK/PD targets used for simulations were different between the two studies. A patient tailored dose based on weight and creatinine CL might reduce variability in teicoplanin AUC and trough concentration (Cmin) values compared with the mgkg^−1^ basis dose (Zhao et al., 2015). Drug monitoring is still recommended.

#### Population Pharmacokinetics of Antifungals in Paediatric Onco-hematological Malignancies

The PopPK studies of antifungals in paediatric onco-hematological malignancies are presented in Tables 3, 4.

**TABLE 3:** Population pharmacokinetic studies of the antifungals Voriconazole and Posaconazole in paediatric onco-hematological malignancies.

	Voriconazole				Posaconazole
Author	Walsh 2004 Walsh et al. (2004)	Karlson 2009 Karlsson et al. (2009)	Muto 2015 Muto et al. (2015)	Gastine 2017 Gastine et al. (2017)	Boonsathorn Boonsathorn et al. (2019)
Study location	United Kingdom/United States/Costa rica/Panama	Europe	Japan	Germany	United Kingdom
Underlying disease (number of patients)	Malignant diseases including	Malignant diseases including	Malignant diseases including	Allogeneic HSCT	Predominantly bone marrow transplant n=86
	- leukaemia n=7	- leukaemia n=56	- leukaemia n=21		
Indication	Prophylaxis or treatment of systemic FI	Prophylaxis of systemic FI	Prophylaxis of systemic FI	Prophylaxis of systemic FI	Prophylaxis or treatment of systemic FI
Number of patients	35	82	21	23	117
Age (years)*mean or **median (range)	*6.2 (2–11)	(2–11)	**10 (3–14)	Age≤12: **8 (0.5–12) Age>12: **14 (13–21)	**5.7 (0.5–18.5)
Weight (kg)*mean or **median (range)	*23.4 (12–54)	*22.8 (10.8–54.9)	**31.5 (11.5–55.2)	Age≤12: **27 (7–44) Age>12: **56 (39–85)	**17.8 (6.05–74.8)
Drug dose (mg/kg)	Single IV doses: 3 to 4	Single IV doses: 3 to 4	Multiple doses: LD 6 to 9 BID IV, followed by MD 4 to 8 BID IV, and then 9 mg/kg or 200 mg BID PO (suspension)	Age≤12: 7 BID IV	Median 13.11 (range, 2.67–48.95) PO (tablets and suspension)
	Multiple IV doses: LD 6 BID followed by MD 3 to 4 BID	Multiple doses: LD 6 BID IV, followed by MD 3 to 8 BID IV, and then 4 to 6 BID PO (suspension)		Age>12: LD 6 BID IV, followed by MD 4 BID IV, and then 200 mg BID PO (suspension)	
PK sampling design	Specific PK study sampling	Specific PK study sampling from 3 studies	Specific PK study sampling	Specific PK study sampling	TDM sampling
Number of samples	355	1274	276	187	338
Software	NONMEM	NONMEM	NONMEM	NONMEM	NONMEM
Number of compartment(s)	2 with linear elimination	2 with Michaelis-Menten elimination	2 with mixed linear and non-linear elimination (model previously developed by Friberg et al. (2012), Hong et al. (2006)	2 with Michaelis-Menten elimination	1
Significant covariate on CL	WT	WT (linear function, noncentred)	WT (Allofixcoef function, 70 kg centred), CYP2C19 genotype status, and age	WT (Allofixcoef function, 70 kgcentred)	WT (Allofixcoef function, 70 kgcentred)
	CYP2C19 genotype status	CYP2C19 genotype status			
	ALT, ALKP	ALT			
Significant covariate on V	WT	WT	WT (Allofixcoef function, 70 kg centred)	WT (Allofixcoef function, 70 kgcentred)	WT (Allofixcoef function, 70 kgcentred)
Significant covariate on suspension bioavailability		None	None	None	Diarrhoea, co-medication with PPI, dose
Covariates tested without significant effect on PK parameters	Age	Age, gender, height, ethnic origin, serum creatinine, ALKP, GGT, albumin, total bilirubin, and total protein levels	Gender, liver function parameters	Age, gender, body surface area, CRP, bilirubin, AST, ALT, GGT, ALKP, and serum creatinine	Age, treatment/prophylaxis, co-medications (other than PPI)
		Co-medications (CYP2C19 inh, CYP2C9 inh, CYP3A4 inh, CYP450 ind), underlying disease, and presence of mucositis			
CL and/or Vmax estimates (typical values)	CL 0.40 L/h/kg	CL 0.582 L/h/kg in CYP2C19 homozygous extensive metabolizers and km 3.03 ng/ml	CL 2.35 L/h and Vmax at 1h 46.1 mg/h	Vmax 51.5 mg/h standardised to a 70 kg individual	Tablet apparent CL 15 L/h standardised to a 70 kg individual
			for a patient weighing 20 kg		
Validation	NA	Internal	Internal	Internal	Internal
PK/PD target(s) used for dosing optimization (simulations)	AUC	AUC: Objective: to achieve similar exposures to those observed in adults receiving approved dosing regimens	AUC: Objective: to achieve similar exposures to those observed in non-Japanese children receiving the same dosing regimen	1) Trough concentrations of 1–6 mg/L	Steady-state trough concentrations of >0.7 mg/L for prophylaxis and >1 mg/L for treatment
	Cmean				
	Objective: to achieve similar exposures to those observed in adults receiving MD 3 mg/kg BID				
				2) AUC/ MIC >32.1	
Dosage recommendation based on results of modelling and simulation	IV: MD 4 mg/kg BID	IV: 7 mg/kg BID (no LD)	- Age [2–12 [ or [12–15 [years and WT < 50 kg: LD 9 mg/kg BID IV for 1day, followed by MD 8 mg/kg BID IV for 6 days, and then MD 9 mg/kg BID PO for 7 days- Age [12–15 [years and WT ≥ 50 kg: LD 6 mg/kg BID IV for 1 day, followed by MD 4 mg/kg BID IV for 6 days, and then MD 200 mg BID PO for 7 days	Age 2–12 years: LD 9 mg/kg TID IV for 3 days, followed by MD 8 mg/kg BID IV	Initial treatment dose: age 6 months to 6 years: 200 mg suspension QID- age 7–12 years and cannot take tablets: 300 mg suspension QID- age 7–12 years and can take tablets: 200 mg tablets TID. Initial prophylaxis dose: age 6months to 6 years: 200 mg suspension TID- age 7–12 years and cannot take tablets: 300 mg suspension TID- age 7–12 years and can take tablets: 200 mg tablets TID
	PO: 200 mg BID (no LD)				

HSCT, hematopoietic stem cell transplantation; FI, fungal infection; IV, intravenous; LD, loading dose; MD, maintenance dose; PO, per os; BID, twice a day; TID, three times a day; QID, four times a day; PK, pharmacokinetic; TDM, therapeutic drug monitoring; CL, clearance; V, volume of distribution; Km, Michaelis Menten constant; Vmax, maximum elimination rate; WT, weight; Allofixcoef function, weight included as an allometric power function using fixed coefficients of 0.75 for CL and 1 for V; 70kgcentred, normalized according to data for a 70kg individual; ALT, alanine aminotransferase; AST, aspartate aminotransferase; ALKP, alkaline phosphatase; GGT, gamma glutamyl transferase; PPI, proton pump inhibitors; CYP, cytochrome; inh, inhibitor; ind, inducer; CRP, C reactive protein; Internal validation: diagnostic plots +/− visual predictive checks +/− bootstrap; NA, not available; PD, pharmacodynamic; AUC area under the concentration-time curve; Cmean, geometric mean concentrations in plasma; MIC, minimum inhibitory concentration.

**TABLE 4:** Population pharmacokinetic studies of dextrose and lipid Amphotericin B (D and L-AmB) in paediatric onco-hematological malignancies.

	D- and L-AmB	L-AmB	L-AmB	L-AmB
Author	Nath 2001 Ayestarán et al. (1996)	(Hong et al. 2006) (87)	Ohata 2015 Ohata et al. (2015)	Lestner 2016 Lestner et al. (2016)
Study location	Australia	Australia	Japan	United Kingdom/United States
Underlying disease (number of patients)	Malignant diseases including	Malignant diseases (not detailed)	Malignant diseases including	Malignant diseases including
	- ALL n=22		- ALL n=71	- HM n=52
	- AML n=19		- AML n=5	
Indication	Suspected or proven FI (Fever/neutropenia)	Suspected or proven FI (Febrile neutropenia)	Suspected or proven FI (e.g., febrile neutropenia	Suspected or proven FI
Number of patients	57	39	39	35
Age (*months or **years) mean ± SD	74.5 (9–190.5)*	7.1 ± 5.1**	8.4 ± 4.5**	8.7 ± 4.6**
Weight (kg) mean ± SD	21.6 ± 10.2	28.8 ± 19.8	27.1 ± 14.1	26.9 ± 14.0
Intravenous drug dose mg/kg/24h	1 (over 2 h)	0.8 to 5.9 (over 1 h)	1 to 5 (over 1–2 h)	2.5 to 10 (over 1 h)
PK sampling design	Specific PK study sampling	Specific PK study sampling	Specific PK study sampling	Specific PK study sampling
Number of samples	581	637	159	NA (7–12 per patient within each sampling period)
Software	PPharm	NONMEM	NONMEM	PMetrics
Number of compartments	2	2	2	2
Significant covariate on CL	WT (Allofixcoef function, noncentred)	WT (exponential function, MEDcentred)	WT (linear function, MEDcentred)	WT (Allofixcoef function, 70 kg centred)
	Method of AmB administration (D- or L-AmB)		
Significant covariate on V	WT (Allofixcoef model, noncentred)	WT (exponential function, MEDcentred)	WT (linear function, MEDcentred)	None
Covariates tested without significant effect on PK parameters	Age, height, gender, diagnosis, history of prior bone marrow transplant, coadministration of total parenteral nutrition, co-medications: acyclovir, cyclosporin, ondansetron, morphine, diuretics, and promethazine	Age, height, and gender	Serum creatinine, BUN, AST	Liver function, serum albumin, white blood cell count, total protein concentrations, use of parenteral nutrition, and concomitant steroids
			ALT, K, Mg, co-medications
CL estimates	D-Amb: *0.038 ± 0.015 L/h/kg	**0.44 L/h for a patient weighing 21 kg	**0.25 L/h for a patient weighing 23 kg	**0.67 L/h standardised to a 70 kg individual
	L-Amb: *0.052 ± 0.021 L/h/kg		
*mean ± SD individual values or **typical value			
Validation	External (26 patients/83 samples)	Internal	Internal	Internal
PK/PD target used for dosing optimization (simulations)	NA	Suggested target: Cmax,ss/MIC (no threshold available)	NA	NA
Dosage recommendation based on results of modelling and simulation	NA	NA	NA	NA

D-AmB, dextrose amphotericin B; L- AmB, lipid amphotericin B; ALL, acute lymphoblastic leukaemia; AML, acute myeloid leukaemia; HM, hematological malignancy; FI, fungal infection; PK, Pharmacokinetic; NA, not available; CL, clearance; V, volume of distribution; WT, weight; Allofixcoef function, weight included as an allometric power function using fixed coefficients of 0.75 for CL and 1 for V; MEDcentred, centred on the median weight of the population; 70kgcentred, normalized according to data for a 70kg individual; BUN, blood urea nitrogen; AST, aspartate aminotransferase; ALT, alanine aminotransferase; K, blood potassium; Mg, blood magnesium; Internal validation: diagnostic plots and bootstrap; PD, pharmacodynamic; Cmax,ss/MIC, peak concentration at steady state over the minimum inhibitory concentration.

In children, voriconazole PK studies demonstrated high IIV with no apparent relationship to dose in immunocompromised children (Walsh et al., 2010; Pieper et al., 2012). Bioavailability is substantially lower in children than in adults with HM (20 and 59.4%, respectively) (Karlsson et al., 2009).

Four PopPK studies in children were analyzed and summarized in Table 3 (Karlsson et al., 2009; Walsh et al., 2004; Muto et al., 2015; Gastine et al., 2017), the larger one being by Karlsson (Karlsson et al., 2009). An additional one was excluded because of missing information (Carlesse et al., 2019). Data were obtained from rich sampling in patients receiving voriconazole in experimental settings in all four cases. Voriconazole was modeled either with linear or non-linear or mixed of both linear and non-linear elimination. The following covariates were significant in 1–4 of the analyzed models: weight, CYP2C19 genotype status, alanine aminotransferase (ALT), alkaline phosphatase (ALKP) and population age groups (adolescent or child). CYP2C19 deficient genotypes and increased levels of ALT were the most important determinants of voriconazole CL, associated with lower CL values. The additional importance of age was evidenced in the recent study by Yan, showing that, in the paediatric population, the patients younger than 3years, might need higher doses to reach the same trough concentrations and exposure than patients over 3years (Yan et al., 2018). Simulations conducted in the four analyzed studies led to the conclusion that the dose required in children was higher than in adults. However, recommended dosages differed from one study to one other for the same age group (Muto et al., 2015; Gastine et al., 2017). Only one Posaconazole PopPK study was conducted in children (Boonsathorn et al., 2019): weight, formulation (suspension or tablet), dose, diarrhea and coadministration of proton pump inhibitors had a significant impact on PK parameters. The estimated values of CL/F and V/F related to the delayed-release tablet formulation and standardized to a 70 kg individual were comparable to those reported in adults. These children showed a higher IIV on CL/F compared to adults (63.0 vs. 24.2 or 37.9%) (van Iersel et al., 2018; Petitcollin et al., 2017) suggesting a potential age-associated maturation of hepatic UGT1A4. Suspension had poor and saturable bioavailability, which decreased with increasing dose. Diarrhea and proton pump inhibitors were also associated with reduced bioavailability of the suspension. Based on the probability of target attainment (PTA) of trough concentration >1mg/L in fungal infection treatment, the authors issued dosage recommendations in children up to 6 years and between 7 and 12 years with an initial dose of 200 and 300 mg suspension four times daily, respectively. When tablets can be used in patients aged seven or over, 200 mg tablets three times daily are required. Dosage have then to be adapted to TDM after the initial phase of treatment.

For amphotericin B, the first PopPK study in children by Nath (Nath et al., 2001) compared D and L-Amb, and then analyzed the two formulations separately (Table 4). This study was conducted with significant number of patients and samples, in a wide range of ages, and showed that weight and formulation (D or L-AmB) had a significant impact on PK parameters. Only one of the four PopPK studies identified resulted in dosage recommendation. Using their previously developed model (Nath et al., 2001), Nath et al. proposed weight-based dosage recommendation for D-AmB (i.e., 1.25–1.5, 1, and 0.75 mg/kg/day for children weighing 10–25, 25–45, and 45–55 kg, respectively) targeting the 0.76–1.05 mg/L range of through level at steady-state (Nath et al., 2007). Lestner and co-authors showed the absence of correlation between absolute dose and exposure (maximum concentration–Cmax, minimum concentration–Cmin, or AUC_0–24_) but a significant correlation between steady-state exposure (AUC_0–24_) and change in serum creatinine. In Japanese paediatric patients (Ohata et al., 2015), the predicted parameters Cmax/dose and AUC_0-24_/dose were similar to those in the non-Japanese paediatric patients (Kohno et al., 2013) and Japanese adult patients at 1.0, 2.5, and 5.0 mg/kg/day given as 1 h infusion.

## Discussion

In this review, we analyzed the population pharmacokinetics of five anti-infectives in paediatric patients with onco-hematological diseases. The studies, based on a nonlinear mixed effects modeling approach, aimed to estimate the typical population PK parameters, their variability between patients, and the variability between occasions and within patients, and to identify the covariates with significant impact on variability in PK.

Most PK studies conducted in children focus on age and organ maturation/function to explain variability in drug disposition. In addition to these key covariates, studies focusing on the potential impact of the disease underlying infection on anti-infective PK are sparse in the paediatric field. Data are missing in many paediatric subpopulations presenting with specific diseases. In paediatric malignancies, most paediatric chemotherapy regimens are intensive with high risk of infection complicating profound neutropenia and requiring anti-infectives at effective and non-toxic doses. We focused our research on the frequently administered glycopeptides (vancomycin and teicoplanin) and antifungals (posaconazole, voriconazole and amphotericin B). Our review shows that data on anti-infectives are limited in children with cancer and that their optimal dosing regimen remains controversial or undefined.

PK and PopPK determine the relation dose/concentration and participate to identify and quantify the impact of covariates on drug disposition. Exposure to anticancer drugs, most of them having a narrow therapeutic range, is central to optimize cure rate of paediatric patients with malignant diseases. However, during treatment, induced immunosuppression is at high risk of infection and anti-infective dosage, if inadequate, may result in infection-related morbidity and increased mortality, making optimization of dosing regimen essential. According to regulatory guidelines, antimicrobial agents are good examples of drugs for which modelling and simulation techniques can be used to develop dosage recommendations in children. PK/PD surrogate markers of efficacy that are used for this purpose include 1) a PK parameter (AUC, Cmax, Time) (Kearns et al., 2003), 2) a PD parameter (MIC) which is function of the germ responsible for the infection, and based either on identification of the germ or more frequently on local germ epidemiology if infection is only suspected.

PK and PopPK of glycopeptides in children with HM diseases are sparse.

IIV in vancomycin disposition in children (neonates excluded) was reported to be primarily linked to patient’s age, type of disease and clinical condition (e.g., renal function, proven infection) (Chang, 1995; Krivoy et al., 1998; Wrishko et al., 2000; Marsot et al., 2012; Hadi et al., 2016; Tkachuk et al., 2018). The impact of malignancy on vancomycin disposition was initially reported by Chang, who showed that vancomycin CL in 33 paediatric patients with malignancy was significantly larger than in 31 patients without cancer while the volumes of distribution were similar (Chang, 1995). The impact of febrile neutropenia was tested in only one study including 109 children with hematological and solid malignancies (Keita et al., 2016), using the model previously developed in adults by Yasuhara (Yasuhara et al., 1998). Multilinear regression analysis of individual patient CL identified age and estimated glomerular filtration rate (eGFR) as covariates affecting drug disposition. Febrile neutropenia did not show any significant impact on CL. Accordingly, in children with malignancy, higher doses than the currently used dosage regimen of 30–40 mg/kg/24 h, are needed to increase the percentage of patients reaching the PK/PD vancomycin AUC/MIC breakpoint of at least 400 h (value determined in adults with *Staphylococcus aureus* pneumonia (Moise-Broder et al., 2004)) or the steady-state target concentration of 20–25 mg/L, while limiting the risk of emergence of vancomycin-resistant microorganisms (Seixas et al., 2016). Optimal doses have to be adapted to weight, creatinine CL, type of disease and co-administration of cyclosporin if any, but remain to be validated prospectively, both in terms of safety and efficacy.

In the teicoplanin PK studies in paediatric malignancy, children had more variability in drug exposures than the adults. The two PopPK studies on paediatric malignancies confirmed that teicoplanin CL was higher in paediatric cancer patients than in children without cancer, with weight and creatinine CL being significant covariates (Ramos-Martín et al., 2014; Zhao et al., 2015). This is most probably related to high glomerular filtration secondary to hyperhydration which is included in HM protocols. In addition, the complex composition of generic teicoplanin products may have a potential impact on both biological analysis and PD. Additional data showed that current weight-based dosage was associated with a low proportion of patients attaining minimum recommended serum drug concentrations at steady state (Cmin value of 10 mg/L) (Dufort et al., 1996; Sánchez et al., 1999; Strenger et al., 2013). According to these data, teicoplanin individualized dosing regimen needs to be recommended for different renal function groups and TDM remains recommended in HM patients.

For antifungals, data are even more limited.

For voriconazole, high paediatric variability is partially explained by body weight, cytochrome P450 2C19 genotype, liver function, and concomitant medications. However, although the genotyping status helps to explain the variability in voriconazole exposure, the CYP2C19 genotyping status alone does not warrant dose adjustment as the voriconazole exposures varied widely within each genotype and overlap considerably across CYP2C19 genotypes. Voriconazole monitoring remains recommended. Therefore, experts advise TDM, in particular in younger children (Chen et al., 2012).

For posaconazole, and according to data obtained in children with malignancy, weight and formulation (suspension or tablet) have an important impact on bioavailability. However, data are extremely limited, did not explore additional covariates already identified in adults such as pharmacogenetic biomarkers and additional studies are particularly needed to validate posaconazole use in paediatric malignancies. Although used in children, this drug is prescribed off-label, as the marketing authorization stated that “safety and efficacy are not established in children aged below 18years” (Table 1).

Amphotericin B is formulated as amphotericin B-deoxycholate (D-AmB) and lipid emulsions (L-AmB). In children, a classical dose escalation study including 40 immunocompromised paediatric patients (2.5, 5.0, 7.5 or 10 mg per kg L-AMB) concluded that L-AMB could be administered to paediatric patients at dosages similar to those of adults but azotemia may develop, especially in those receiving ≥5.0 mg/kg/day (Seibel et al., 2017). In children, Lestner and co-authors (Lestner et al., 2016) showed the absence of correlation between absolute dose and exposure (Cmax, Cmin, or AUC_0–24_) but a significant correlation between steady-state exposure (AUC_0–24_) and change in serum creatinine. Weigt-based dosage recommendation to reach the target through level at steady-state were issued for D-AmB but not for L-AmB (Nath et al., 2001; Nath et al., 2007). When immunocompromised children experience fever that persists in spite of broad-spectrum antibiotic therapy, they receive D-AmB by the standard dose of 1 mg/kg/day that may be insufficient to prevent fungal surinfection or to control clinically detected or undetected fungal infection. Here again, additional PK and efficacy studies are required for a safer use in cancer children.

As illustrated by the present review, PopPK studies on antibiotics and antifungals including in paediatric malignancy are limited for well-known reasons, ethical and technical. The major barriers to paediatric PK studies are the relatively large volumes of blood loss during the study period, difficulty in timing of PK samples due to the critical clinical condition and a relatively low rate of informed parental consent (Baker et al., 2018). For this reason, many drugs are used off-label and enter the paediatric care protocols because clinicians perceive them to have a more useful spectrum of activity and/or better profile of tolerance than the currently used anti-infectives.

Population PK allows to determine PK parameters with a formal PK design with planned (pre-selected) sampling times, with opportunistic samples or a combination of planned and opportunistic samples (Leroux et al., 2015). In our review, it should be noted that, in most PopPK studies, sometimes retrospective and based on TDM, the number of patients was limited (lower than 100), and age range and malignant underlying disease were variable. Only a few studies performed a meta-analysis of data from different studies, as previously done in neonates (Jacqz-Aigrain et al., 2019) allowing to combine sufficient data to reach a larger number of patients, increase study power and identify covariates.

Most studies used a nonlinear mixed effects mathematical method, estimating PopPK parameters (CL and V) and their variability, based on the significant impact of covariates. Covariates in the context of paediatric malignancy include age, weight, organ maturation and function, but also other determinants such as underlying disease groups, comedications, and pharmacogenetics. Allometric scaling is an empirical examination of the relationships between the PK parameters and size (body weight). Allometric power parameters are often fixed at values of 0.75 for CL and 1 for distribution volume on the basis of physiologic consideration of size impact on metabolic processes (Anderson et al., 1997; Anderson and Holford, 2011). As shown here, the allometric coefficients need to be estimated in a limited number of cases (Johnson, 2008). The covariates renal function (reflected by creatinine or creatinine CL), hepatic function (reflected by ALT and ALKP) and pharmacogenetics were frequently tested. In the case of voriconazole, CYP2C19 genetic polymorphism, identified in adults affecting voriconazole disposition was not identified as a significant contributor to variability in children. As illustrated with this example, the role of pharmacogenetic biomarkers in variability may not be significant when the number of patients of deficient metabolizer genotypes is low, when PK overlap exists between the different genotypes and/or when genotype expression did not reach maturation (Lestner et al., 2016).

Once the PK model is developed, internal validation (using the same dataset) and external validation (requiring additional independent patients) are required. In most paediatric studies, validation was internal, predominantly based on goodness of fit plots and bootstrapping. In the studies that we analyzed, external validation was the exception, although it is more stringent.

Simulations of dosing regimens based on the validated model aim to inform optimal dosing in children that achieves target exposure comparable to that of adults. Of note, the adult PK/PD target thresholds do not take into account developmental aspects of immunocompetence; indeed, the immune system gradually changes during infancy to mature and expand during growth and to respond efficiently to acute infections (Anderson and Holford, 2011; Friberg et al., 2012). In addition to reduced immunocompetence due to incomplete immune maturation, the role of therapeutic immunosuppression would require to be explored. As illustrated in this review, different PK/PD targets may be used for dosing optimization of the same drug, with a lack of consensus regarding which target is optimal for this purpose. Efforts should be made to further explore this issue.

Before implementation of the new dosing regimen into the clinics, validation of exposure, safety, and tolerability in a carefully designed clinical trial will be needed. However, for most if not all studies, the clinical validation is not available, although response to anti-infectives is known to depend not only on drug exposure but also on age, associated therapies and type of disease.

## Conclusion

In conclusion, many antibiotic and antifungal compounds are not approved for children or their optimal dosage is unknown, although differences in drug disposition may be anticipated in children compared to adults. We showed that PopPK data of the frequently prescribed glycopeptides and antifungals are very limited in children, although they are prescribed in most patients with hematologic malignancy. A few inform variability in disposition, identify significant impact of weight and additional covariates (organ function, disease subgroups) and led to dosage recommendations taking into account the identified variables. This review highlighted the lack of consensus regarding PK/PD targets used for dosing optimization, and regarding dosage recommendations when available. Additional PopPK and PK/PD studies are needed in this specific population of patients. In addition, clinical studies should be performed to prospectively validate the dosing regimens adapted to infection in paediatric patients with malignancy.
